# Resection of a large thoracolumbar intradural ependymoma: a 2D operative video

**DOI:** 10.3171/2023.7.FOCVID2378

**Published:** 2023-10-01

**Authors:** Daniel M. Aaronson, Brandon Laing, Randall Treffy, Saman Shabani

**Affiliations:** Department of Neurosurgery, Medical College of Wisconsin, Milwaukee, Wisconsin

**Keywords:** spinal cord tumor, ependymoma, microsurgery

## Abstract

In this video, the authors present the resection of a large thoracolumbar intradural ependymoma in a 33-year-old female. The patient underwent T9–L3 laminectomies, intradural tumor resection, and posterior instrumented fixation and fusion. The surgical procedure aimed to relieve the mass effect, obtain a diagnosis, prevent further neurological decline, and achieve a potential curative resection. The pathology confirmed a myxopapillary ependymoma, a rare tumor with a preference for the conus medullaris, cauda equina, or filum terminale. The video provides insights into the case, surgical steps, clinical outcomes, and background information on myxopapillary ependymomas and treatment options.

## Transcript

### 0:25 Case Presentation.

The patient is a 33-year-old female who presented with 2 months of lower-back pain, right leg weakness, difficulty with ambulation, and intermittent bilateral lower-extremity paresthesias after a recent motor vehicle accident.

On exam, the patient was confirmed to have right lower-extremity weakness, 2/5 proximally and 3/5 distally, with intact motor strength in her remaining three limbs.

She was also found to have abnormal sensation to light touch and proprioception throughout her right lower extremity in a nondermatomal distribution, as well as increased tone with decreased patellar tendon reflex in that same right lower extremity.

### 1:00 Neuroimaging Findings.

An MRI of the thoracolumbar spine was obtained demonstrating a longitudinally extensive enhancing intradural lesion from the level of T9 to the inferior aspect of L4. The lesion causes significant compression of the lower thoracic spinal cord parenchyma with T2 and STIR hyperintensity extending from T4/5 through T9/T10, as well as peripheral displacement of the cauda equina nerve roots within the thecal sac.

Given the patient’s symptoms and presence of a mass lesion on imaging, the patient was recommended T9–L3 laminectomies and intradural tumor resection with a T9–T11 transpedicular approach and T8–L4 posterior instrumented fixation and fusion. The main goal of the procedure was to relieve the mass effect of the tumor on the spinal cord, especially in light of the patient’s presentation with a neurological decline. The potential benefits of the procedure included obtaining a diagnosis, preventing worsening neurological decline due to further tumor growth, and the potential for a curative resection. Risks of the procedure, in addition to common surgical risks, included lower-extremity paralysis; sensory, bowel, or bladder disturbance; adjacent-segment disease; wound complication or cerebrospinal fluid leak; and tumor recurrence.

Alternatives included observation, radiation, or chemotherapy treatment. However, none of these options would allow for obtaining a tissue diagnosis, nor would the mass effect of the tumor be relieved. Furthermore, treatments such as chemotherapy are not a mainstay treatment for pathologies such as ependymoma. T9–L3 laminectomies and tumor resection without fusion may also be considered. However, in this case, given the ventral extension and size of the tumor, a transpedicular approach as well as multilevel facetectomies were needed to achieve gross-total resection and adequate thecal sac decompression. Thus, a fusion was needed to avoid long-term instability.

### 2:57 Positioning and Necessary Equipment.

In terms of positioning, preflip SSEP and MEP baselines were obtained. The patient was then flipped prone on a Jackson table and a midline incision was planned. The entirety of the thoracolumbar spine was prepped and draped in the usual fashion.

Surgical equipment included the operative microscope, microsurgical instruments, ultrasound, an ultrasonic surgical aspirator, neuromonitoring, a high-speed drill, spinal instrumentation hardware, and allograft.

### 3:23 Key Surgical Steps.

In terms of surgical steps, a midline incision was made, monopolar electrocautery was used to open the soft tissue and fascia with subperiosteal dissection out to the transverse process at each level with preservation of the most cranial (T7/8) and caudal (L4/5) facet joints. Laminectomies from T9 to L4 to have access to both the cranial and caudal extent of the tumor were performed. Facetectomies were performed at T9/10, T10/11, T11/12, T12/L1, L1/2, L2/3, and L3/4 for further decompression. Transpedicular decompression at T9, T10, and T11 due to ventral positioning of the tumor was then performed. The ultrasound was then used to evaluate whether the amount of dural exposure would be sufficient to reach the top and bottom poles of the tumor. Durotomy was performed in a piecemeal fashion from T9 to L4. Microsurgical dissection of the tumor from the nerve roots was followed by microsurgical resection of the tumor utilizing a combination of sharp dissection, and ultrasonic aspiration for tumor debulking followed by dissection off of neural elements. A dural closure was then completed, followed by posterior instrumentation and preparation for arthrodesis utilizing autograft and allograft from T8 to L4. Multilayer closure after dural graft placement over the durotomy site was performed.

### 4:36 Initial Tumor Dissection.

Here we see after dural opening blunt dissection of the tumor taking care to separate the cauda equina nerve rootlets from the tumor tissue. Microsurgical instruments such as a Rhoton 6 can be useful for this maneuver. Patties are carefully placed to delineate already dissected elements. Sharp dissection is also often necessary. Here we demonstrate this technique using an 11 blade. Similarly, microsurgical scissors can also be used. Careful constant instillation of saline via a soft angiocatheter tip can be used to perform hydrodissection as well.

A portion of tumor tissue was then removed to send for a frozen pathology specimen. Further blunt dissection was then performed until ventral dura was clearly identified.

### 5:37 Tumor Debulking.

An ultrasonic surgical aspirator was then utilized to debulk the tumor, with bipolar electrocautery used to control bleeding. The durotomy was then carried superiorly using a combination of a Woodson elevator and an 11 blade, followed by hydrodissection on the lateral surfaces to free the cord from dural adhesions. Tumor removal was then continued and carried to the level of the conus, and the gutters and dural elements were inspected for residual tumor. The entirety of the dural exposure can be seen here from the inferior thoracic cord, down to the lower lumbar rootlets.

The dura was then closed in a watertight manner using 5-0 Prolene suture on a VB-1 needle. This was followed by an overlay dural graft with spinal sealant. No CSF egress was noted after a Valsalva maneuver.

### 6:45 Review of Clinical Outcome.

The patient tolerated the procedure with no apparent complications. Pathology demonstrated a WHO grade II myxopapillary ependymoma. At 3 months postoperatively, the patient denied any new neurological deficits with slow improvement of her right lower-extremity weakness and paresthesias. Postoperative imaging demonstrated gross-total resection of the lesion.

### 7:09 Disease Background.

Ependymomas are a rare CNS tumor that arise from ependymal cells in the central canal, filum terminale, choroid plexus, or white matter near the ventricular surface. Ependymomas are classified by the World Health Organization as grade I, II, or III based on histological findings.^1^

Myxopapillary ependymomas are a rare subset of ependymoma classified as WHO grade II. Myxopapillary ependymomas have an incidence in the United States of around 1.00 per million person-years and are typically found in the conus medullaris, cauda equina, or filum terminale.^2,3^ There are two incidence peaks, one in early adulthood between 25–29 years of age, and another between 45–49 years of age.

Gross-total resection, and if possible, with an intact tumor capsule, are the historical and modern treatment modalities of choice and associated with the lowest recurrence rate.^4^ Although adjuvant radiation is controversial, there appears to be some increase in progression-free survival with adjuvant radiation therapy.^5^

## Disclosures

The authors report no conflict of interest concerning the materials or methods used in this study or the findings specified in this publication.

## Author Contributions

Primary surgeon: Shabani. Assistant surgeon: Aaronson. Editing and drafting the video and abstract: Aaronson, Laing, Treffy. Critically revising the work: all authors. Reviewed submitted version of the work: all authors. Approved the final version of the work on behalf of all authors: Shabani. Supervision: Aaronson.

## Supplemental Information

### Patient Informed Consent

The necessary patient informed consent was obtained in this study.
